# The Influence of Recognition and Social Support on European Health Professionals' Occupational Stress: A Demands-Control-Social Support-Recognition Bayesian Network Model

**DOI:** 10.1155/2017/4673047

**Published:** 2017-11-09

**Authors:** Susana García-Herrero, Jose R. Lopez-Garcia, Sixto Herrera, Ignacio Fontaneda, Sonia Muñoz Báscones, Miguel A. Mariscal

**Affiliations:** ^1^Escuela Politécnica Superior, University of Burgos, Avda. Cantabria s/n, 09006 Burgos, Spain; ^2^Department of Applied Mathematics and Computer Sciences, University of Cantabria, Santander, Spain

## Abstract

Healthcare professionals undergo high levels of occupational stress as a result of their working conditions. Thus, the aim of this study is to develop a model that focuses on healthcare professionals so as to analyze the influence that job demands, control, social support, and recognition have on the likelihood that a worker will experience stress. The data collected correspond to 2,211 healthcare workers from 35 countries, as reported in the sixth European Working Condition Survey (EWCS). The results obtained from this study allow us to infer stress under several working condition scenarios and to identify the more relevant variables in order to reduce this stress in healthcare professionals, which is of paramount importance to managing the stress of workers in this sector. The Bayesian network proposed indicates that emotional demands have a greater influence on raising the likelihood of stress due to workload than do family demands. The results show that the support of colleagues, in general, has less effect on reducing stress than social support from superiors. Furthermore, the sensitivity analysis shows that, in high-demand and low-control situations, recognition clearly impacts stress, drastically reducing it.

## 1. Introduction

Currently, according to the latest studies surveyed, it has been discovered that healthcare professionals undergo high levels of occupational stress as a result of different factors related to the working conditions attributed to their trade [1]. In particular, nurses experience the highest level of stress when compared to other healthcare professionals [2–4].

Stress has been defined in different ways throughout history. Selye [5], one of the pioneers in developing the concept, defined stress as* “The non-specific response of the body to any demand, whether it is caused by, or results in, pleasant or unpleasant conditions”* [5, 6].

The human body's response to stress is exhibited through the appearance of health problems, symptoms, and advanced stages of disease. Many studies analyze the interrelationship between occupational stress and high blood pressure and coronary heart disease [7–13]. Other studies reveal the relationship between stress and musculoskeletal problems [2, 14–16]. At the same time, occupational stress might be the cause of severe depression [17, 18] and insomnia [19–22], which itself conditions and promotes the labor absenteeism of those workers who suffer from it [23, 24].

Various theoretical models have been developed to explain different situations pertaining to the relationship between work and its effect on workers' mental health. Firstly, the most commonly used models are those by Karasek [25], the Job Demand-Control (JDC) model, and the expanded model, Demand-Control-Support (DCS) [26, 27]. The classic model (JDC) by Karasek [25] explains the negative health effects on workers if they do not have sufficient control over their work in relation to labor demands. In Karasek and Theorell's [26] extended model (DCS), high levels of stress are detected when the demands placed on the worker are not in accordance with the worker's own control and decision-making power, and the support received from superiors and/or companions is insufficient. Secondly, the Effort-Reward Imbalance at Work (ERI) model is another commonly used method, developed by Demerouti et al. [28], which deduces that the employees who are most likely to be stressed are those whose efforts are not rewarded or recognized by their organization, in other words, high-effort and low-reward. Thirdly, the Job Demand-Resource (JD-R) model, proposed by Demerouti et al. [28], explains the interactions between labor/work demands and resources to justify high levels of stress in workers.

As indicated above, these models have been used to study occupational stress. For example, Karasek's [25] model (DC) has been used to analyze, by gender and geographical area, the stress exhibited by workers. An analysis of the data collected through this model reveals that differences in working conditions, which include labor demand per worker, as well as the worker's control over his/her own actions in the workplace, determine stress levels [29]. Another example is presented by Kornitzer et al. [30], which is based on utilizing the (JDC) model as a predictor of acute coronary events. The DCS model provides information on the levels of stress for self-, privately, and publicly employed individuals [31].

However, some authors have expanded this further. For example, these models are used as the foundation in the work done by Giauque et al. [32], which expands and reinforces the JD-JR model to include the motivational factor in public services [31] and explains how social reinforcement and support, job recognition, and positive feedback decrease the levels of stress perceived by a public service worker. Salas et al. [33] employed the DCS model in addition to studying work demands, work control, and social support at work and incorporated workplace exposure to violence and bullying as variables to measure stress at work. The ERI model has also been applied outside work life [34], for example, to household and family work [35, 36].

Likewise, other authors have used several of these models in combination. Tsutsumi and Kawakami [37] suggest that the two models (JDC and ERI) are complementary, and we find several examples along these lines [38, 39]. Symptoms of depression have been studied by Dragano et al. [18] through the application of JDC and ERI. Also, Bauer and Groneberg [40] demonstrate with the ERI and JDC models that unfavorable working conditions have a negative effect on the mental and physical health of employees. They also suggest adapting the working conditions to match workers' expectations, the main aim being to lower stress and improve job satisfaction. Also, the study reported by Yu et al. [41] uses a combination of the DCS and ERI models to analyze work absenteeism (due to back pain) caused by psychosocial risks.

There are also other factors unrelated to one's job that condition occupational stress. Let us first consider the family-work conflict. For example, the work by Pal and Saksvik [42] analyzes the relationships between the following variables: work-family conflict (WFC), family-work conflict (FWC), job demands, job control, social support, flexibility in working hours, and job stress, yielding different results for nurses and doctors by country. The work by du Prel and Peter [43] also studies the work-family conflict (WFC) and its relationship to work stress. The Copenhagen Psychosocial Questionnaire [44] was used to measure WFC in their investigation. Secondly, we consider the influence of emotional demands on psychosomatic health complaints. In their work, De Jonge et al. [45] evaluate Karasek's demand-control model by incorporating various demand factors, including emotional demands.

In regard to the methodologies used to carry out this type of analysis, the classical methods predominate: descriptive statistics, inferential statistics, bivariate linear regression, multiple linear regressions, ANOVAS, multiple logistic regression, hierarchical linear regression, multivariate logistic regression, and so on. However, on the one hand most of these techniques are not able to describe the complex, direct/conditional, and linear/nonlinear relationships between the variables considered in the model; and on the other hand, models obtained with other nonlinear (e.g., neural network) or more complex (e.g., multilevel/hierarchical path analysis, Shipley [46] and Peter et al. [47]) techniques are very difficult to interpret and do not yield conclusions about the sensitivity of the target variable(s) with respect to the explanatory variables. Bayesian networks (BN), which have also been widely employed in the field of healthcare/medicine [48–50], are a Probabilistic Graphical Model [51] based on a directed acyclic graph (DAG) that is able to describe both direct and conditional dependences between variables and is easily interpretable by means of the graph or the conditional probabilities obtained.

It should be noted that the use of the Bayesian network methodology has been gradually employed in other areas of knowledge. In the field of occupational health and safety, the one that concerns us, there are several examples of the application of Bayesian networks. As examples, Zhou et al. [52] proposed a model to analyze the influence of climate safety factors and personal experience on human behavior; Yang et al. [53] evaluated the relationship between psychological factors and successful smoking cessation; Akhtar and Utne [54] calculated the risk of human fatigue in maritime accidents; García-Herrero et al. [55] analyzed the influence of working conditions on occupational accidents; Moret-Tatay et al. [56] examined the relationships, in terms of probabilities, between gender, coping, and mental health.

Our study focuses on workers within the healthcare sector and uses Bayesian networks to analyze the probability of working stress occurring based on psychosomatic health complaints. To this end, our model uses concepts from different, widely used models such as the Demand-Control-Support (DCS) model, the ERI model, the Job Demand-Resource (JD-R) model, the emotional demands proposed by De Jonge et al. [45], and the family-work conflict proposed by Pal and Saksvik [57]. Lastly, the demographic variables of age and gender are considered to further analyze their effect on the likelihood of the occurrence of occupational stress.

This work considers the hypothesis that worker stress as measured through psychosomatic health complaints depends on a combination of different factors, such as emotional, family, and work demands, control (i.e., autonomy at work), social support (from colleagues and superiors), and the recognition given to workers. The sensitivity analyses generated with the Bayesian network proposed consider all of the model's variables such that every variable is involved in every analysis.

Specifically, our study answers the following questions:

To what extent is the likelihood of stress caused by workload modified by emotional and family demands?

To what extent does the social support of coworkers or superiors influence the likelihood of stress associated with job demand and control?

To what extent does recognition influence the likelihood of stress associated with job demand and control?

By gender and age, to what extent does recognition influence the likelihood of stress associated with job demand and control?

## 2. Data and Methods

### 2.1. European Working Conditions Survey (EWCS)

The European Foundation for the Improvement of Living and Working Conditions (EUROFOUND) is a three-party European Union Agency (a union between governments, trade unions and employees), established in 1975, whose objective is to provide the knowledge required to contribute to the creation and design of better living and working conditions, as well as to improve policies regarding the working environment. It includes the 28 countries that now comprise the European Union, with the addition of Norway, Turkey, Montenegro, Serbia, Albania, Switzerland, and the former Yugoslav Republic of Macedonia [58], for a total of 35 countries involved.

The European Working Condition Surveys (EWCS) are a source of information on working conditions and the quality of work and employment in Europe, carried out by EUROFOUND. They have been conducted from 1990 to the present. This survey reveals long-term trends in working conditions and considers further aspects, such as employment status, laws regarding working hours, job organization, job-related learning and training, the risk factors involving physical or psychosocial damage, worker health and safety, the worker's involvement in decisions regarding the improvement of working conditions, and their work-life balance and their income and financial security [59].

The data collected in this study was provided by the sixth European Working Condition Survey (EWCS) [60], carried out in 2015 [61], and later published in 2016. It is based on the data extracted from the interviews conducted with 43,850 workers from the 35 different countries mentioned earlier and seeks to capture the versatile aspects of working in today's Europe. The number of interviews conducted in each country ranges from 1,000 to 3,300, depending on the number of inhabitants of working age in each country, with all respondents chosen completely at random [62]. The interviews use 106 questions.

Our study focuses on the healthcare area and, as a result, considers the data from a subgroup of the general sample corresponding to 2,211 workers belonging to this sector, as identified by the “International Standard Classification of Occupations” activities code (ISCO 08-COM). For this research, the workers in the health areas defined in ISCO-08 classification will be considered: health professionals and health associate professionals.

The average age of the respondents was 43, and 79% of them were women. As for countries, the best represented was Belgium, with 172 respondents, while Greece only had 20. Only four countries had over 100 respondents (Belgium, Spain, Germany, and Sweden).

As for the educational level of the respondents, this is shown in Table 1, with most respondents having completed upper secondary education.

### 2.2. Conceptual Model

The conceptual model of the study presented explains how healthcare professionals who are assigned a high number of tasks, with emotional and family demands, who cannot exert any control over their jobs, who have low levels of support from their coworkers or direct supervisors and with low recognition will be more likely to develop occupational stress as measured by psychosomatic health complaints. The model will also explain the results (probabilities) of occupational stress corresponding to the different combinations of these parameters.

The model was designed based on previous studies. First, the “stress” objective variable is measured using the physical symptomatology exhibited by the worker. Authors like Nixon et al. [63] indicate that physical symptoms are physical manifestations that reflect occupational stress factors. Second, the model considers demands, control, social support, and recognition.  Table 2 shows a comparison of the questions used in this study against those used in the original models.

Regarding the demands, it is important to note that the model proposed includes family demands (as proposed by du Prel and Peter [43]) and emotional demands (as proposed by De Jonge et al. [45] in their studies), in addition to any existing job demands. In terms of social support, the model does not work with a single social support variable received by coworkers and direct supervisors. Rather, it presents different variables for each case. In this way the model will consider how support from peers or superiors decreases the likelihood of occupational stress. The control variable expresses the decision authority or autonomy of the worker, as indicated in the DCS model. In addition, the model presented includes the recognition received by the worker for work already performed. Recognition, as the ERI model indicates, explains how workers are most likely to be stressed when their efforts are not rewarded or recognized by their organization [64].

Finally, the model also includes two demographic variables: age and gender. Having these variables in the model makes it possible to differentiate the likelihoods of occupational stress by gender and age, while also taking into consideration different cases, for example, job demand, control, and recognition.

The questionnaire of the 6th EWCS contains 106 questions, from which the variables that will be the object of the study are extracted and with the worker's “stress” arising from the work they perform considered as an objective variable. Specifically, we will analyze the influence of nine variables selected from the general basis, arranged into five groups at the same time:

1. Demographic: gender and age.
2. Demands: emotional demand, family demand, and job demand.
3. Control.
4. Social support: social support from colleagues and social support from superiors.
5. Recognition.

### 2.3. Study Variables

The variables chosen for this study are stress, gender, age, emotional demands, family demands, job demands, control, social support from colleagues, social support from the boss, and recognition. All of them are described in detail below.  Table 3 shows the frequencies of the chosen variables.

#### 2.3.1. Stress

This variable was obtained from question Q78,* “Did you have any of the following health problems?”* In it the following symptoms are listed: hearing problems, skin problems, backache, muscular pains, headache, anxiety, and overall fatigue.

In order to compose the variable, a worker is considered to suffer from stress when he/she has three or more of the above symptoms. These items form a coherent Cronbach's alpha (*α* = 0.65) scale. Thus, the stress variable exhibits two states: no (1) and yes (2).

#### 2.3.2. Gender

The gender variable corresponds to question Q2-a in the EWCS. The variable maintains the two original states: man (1) and woman (2).

#### 2.3.3. Age

This variable was constructed by grouping the ages of the Q2-b question into four states: younger than 35 years of age (1), between 35 and 45 (2), from 45 to 55 (3), and older than 55 (4).

#### 2.3.4. Emotional Demands

Emotional demands are obtained from question Q30-h,* “Please tell me, using the same scale, does your main paid job involve being in situation that is emotionally disturbing for you?”* The original response scale has the following states: all of the time, almost all of the time, around 3/4 of the time, around half of the time, around 1/4 of the time, almost never, and never. The variable is constituted by grouping these states into the following three categories: always or almost always (1), sometimes (2), and almost never or never (3).

#### 2.3.5. Family Demands

Question Q45-d* “How often in the last 12 months have you found it difficult to concentrate on your job because of your family responsibilities?”* was used to establish the family demands variable. The answers have the following states: always, most of the time, sometimes, rarely, and never. The variable is defined in the following three categories: always or sometimes (1), rarely (2), and never (3).

#### 2.3.6. Job Demands

Job demands are obtained from questions Q49-a* “Does your job involve working at very high speed?”* and Q49-b* “Does your job involve working to tight deadlines?”* The original response scale has the following states: all of the time, almost all of the time, around 3/4 of the time, around half of the time, around 1/4 of the time, almost never, and never.

Cronbach's alpha in relation to these two questions is *α* = 0.73. Therefore, the variable is constituted by the rounded mean of the answers obtained from these two questions and is accordingly assigned into the following three categories: always or almost always (1), sometimes (2), and almost never or never (3).

#### 2.3.7. Control

The information pertaining to the control topic was established based on the following questions: Q54-a* “Are you able to change your order of work?”* and Q54- C* “Are you able to choose or change your speed or rate of work?” *Cronbach's alpha for these two questions is *α* = 0.67. Both answers to these questions are summarized in two maxims: yes (1) and no (2).

#### 2.3.8. Social Support from Colleagues

The social support of peers corresponds to question Q61-a* “For each of the following statements, please select the response which best describes your work situation. Your colleagues help and support you?”* The answers have the following states: always, most of the time, sometimes, rarely, and never. The variable is defined in the following three categories: always or sometimes (1), rarely (2), and never (3).

#### 2.3.9. Social Support from the Boss

The variable concerning the social support provided by the direct supervisors is based on questions Q61-b and Q63. Q61-b asks,* “For each of the following statements, please select the response which best describes your work situation. Your manager helps and supports you?”* Q63 asks,* “To what extent do you agree or disagree with the following statements? Your direct supervisor respects you (a), gives you praise and recognition when you do a good job (b), is successful in getting people to work together (c), is helpful in getting the job done (d), provides useful feedback on your work (e), encourages and supports your development (f).”*

These seven questions have five possible answers: strongly agree, tend to agree, neither agree nor disagree, tend to disagree, and strongly disagree. Cronbach's alpha for these answers is *α* = 0.88, and this variable has been grouped and classified into three categories: agree, neither agree nor disagree, and strongly disagree.

#### 2.3.10. Recognition

The “recognition” variable corresponds to question Q89-c: “I receive the recognition I deserve for my work.” The variable originally featured five responses: strongly agree, tend to agree, neither agree nor disagree, tend to disagree, and strongly disagree. For this study the following three categories were used: agree, neither agree nor disagree, and strongly disagree.

### 2.4. Bayesian Networks

**Figure 1:**
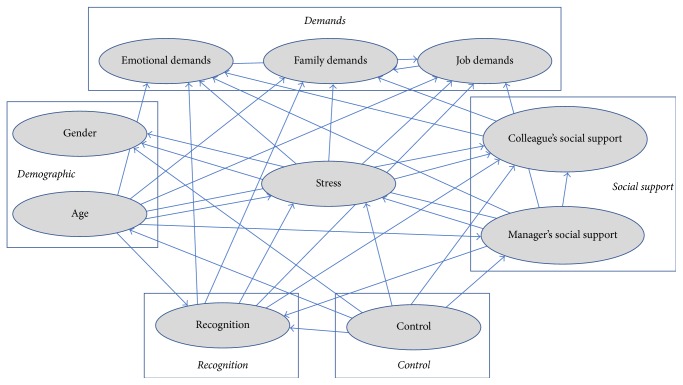
Bayesian network graph.

Probabilistic Graphical Models [51] combine graphs and probability theories to efficiently learn the joint probability distribution of a multivariate problem. The graph of the model (see Figure 1) describes dependence (conditioned or not) relationships between variables that are used to both simplify the factorization of the joint probability distribution and to use knowledge of several variables to predict the state of the result.

Bayesian networks [65] are a type of PGM based on directed acyclic graphs (DAG). In this case, the joint probability distribution can be factored in terms of conditioned probabilities:

(1) $$\begin{array}{c} p\left(\;x_{1},x_{2},\ldots ,x_{n}\;\right)=\overset{n}{\underset{i=1}{\prod }}p\left(\;x_{i}\;|\;\pi_{i}\;\right), \end{array}$$

where *π*_*i*_ corresponds to the parents of *x*_*i*_.

Based on the data, both graph and probabilities can be automatically learned [66] following a two-step process. First, the DAG is obtained by searching for the optimal compatible structure of dependence/independence relationships between variables (structural learning), and then the probabilities are obtained based on the factors defined by the DAG (parametric learning).

The factorization, together with the graph, is used to infer, based on any evidence or new knowledge about the state of one or several variables, the probabilities of the remaining variables by applying efficient algorithms.

### 2.5. Model Performance: Receiver-Operating Characteristics

In order to evaluate the model obtained by applying Bayesian networks to the data, a cross-validation approach was considered to avoid model overfitting. In our case, a 10-fold cross-validation was developed to define 10 nonoverlapping data subsets from the full sample, each of them containing N/10 elements. Each data subset was used as a test set, with the remaining data used in each case as a training set to adjust the Bayesian network. Thus, a prediction of the whole sample was obtained from independent test samples joining the 10 fold's prediction and evaluated in terms of the Receiver-Operating Characteristic (ROC) curve [67], which is a standard validation approach for probabilistic and binary classifiers and, more specifically, the area under curve (AUC), which varies from 0.5 (random guess) to 1 (perfect performance) and can be interpreted as a measure of overall accuracy [68]. An AUC of 0.85 was finally obtained for the Bayesian network, reflecting the high skill of the model.

## 3. Results and Discussion

### 3.1. Network Graph

The Bayesian network proposed according to the above criteria provides an acyclic directed graph, as shown in Figure 1. On this graph we can see the different relationships between the various variables and the resolving influence that some variables have on others.

### 3.2. Sensitivity Analysis: Job Demand, Family Demand, and Emotional Demand versus Stress

We first conducted a sensitivity analysis of the demands that workers face over the course of their jobs (see Table 4) in order to determine how family and emotional demands affect the probability of work stress produced by occupational demands.

The results indicate that the probability of stress in a worker due to occupational demands increases slightly when coincident with elevated family demands, going from 51.5% to 52.3%. When the occupational demand is low, however, family demands generate a significant increase in the likelihood of stress, going from 29.3% to 41.0%. The effect of the demands arising from emotional situations on the job, added to occupational demands, raises the probability of suffering from occupational stress by nearly 10%. It is worth noting that an effective variable for buffering the stress caused by high occupational demands is the variable associated with emotional situations, more so than the family variable, with a low emotional demand reducing the initial stress by 14.1%.

### 3.3. Sensitivity Analysis: Job Demand, Control, and Social Support versus Stress

This sensitivity analysis considers, first of all, the job demand and control variables and their effect on the appearance of stress. The results for the probability of job stress considering these variables are shown in the third column of Table 5. Note that the stress probability values are highest for the most unfavorable combination, that is, high job demands and low control, reaching 52.8%. As labor demands decrease, the probability of stress, in turn, diminishes. For example, if job demand is low and control is high, the probability of stress drops drastically, by 24.1% (from 52.8% to 28.6%).

The variables related to social support are then added as evidence. Initially, we consider the case involving support from colleagues. If we analyze the same case, we see an increase in the likelihood of stress when there is a lack of support from colleagues, giving rise to a range spanning 20.8% (from 52.8 to 73.6%). If the situation changes as concerns colleague support, this initial value drops slightly by 3.4% (52.8%–49.3%). When circumstances are positive for the worker, meaning low demand, high control, and colleague support, the likelihood of experiencing stress is practically unchanged (28.6%–29.8%).

Secondly, we analyze the influence of social support from superiors at three levels (from strongly agree to strongly disagree). This shows a clear tendency toward an increased likelihood of stress as this support decreases. As we see in Table 5, in the worst scenario, the initial probability is 52.8%, which rises by 41.3% when the worker does not receive any social support from his/her superior. In the opposite case, that is, when the worker does receive this support, the initial probability drops by 7.7%, to reach a value of 45.0%.

In conclusion, we see that social support from peers has less effect on reducing the likelihood of suffering work-related stress than support from superiors, and this lack of social support increases the values obtained to a lesser extent.

### 3.4. Sensitivity Analysis: Job Demand, Control, and Recognition versus Stress

Considering once more the variables involving job demand and control and adding the possible influence of job recognition or recognition, we see that this last variable has a more significant effect on the likelihood of experiencing work-related stress. The values shown in Table 6 indicate that in a situation created by high demands and minimum control, the reward variable is clearly influential.

When an employee's work is recognized, the likelihood that he/she will experience stress is lowered by 22.1% (52.8%–30.7%), whereas if his/her work is not, it rises by 16.7% (52.8%–69.4%). Low job demands and positive control over one's job, as well as recognition of a job well done, lower the probability of stress to 21.2%, whereas a lack of recognition raises the probability up to 51.2%. In summary, the influence of the reward variable accounts for a significant change, both lowering the probability of stress, when one's work is recognized, and increasing it, when it is not.

### 3.5. Sensitivity Analysis: Job Demand, Control, and Recognition versus Stress—Demographic Analysis

Starting from the previous analysis, which presents the job demand, control, recognition, and stress variables, we shall now add a consideration of gender and age to see if there is any change in the probability between men and women, as well as the influence of the various age ranges. This analysis will focus on finding the possibilities that work-related stress is present in extreme situations, that is, negative and positive conditions pertaining to job demands and job control. These will be differentiated by gender after including the recognition variable and the ages of the workers surveyed.

The results show, first of all, that in every case men are less likely to experience stress than women (Table 7). Our goal, however, is to analyze the influence that the recognition variable has on stress levels in men and women. While a man in the extreme situation of high job demands and low control with no recognition is 58.6% likely to feel stress, this figure drops by 37.9% (from 58.6% to 20.7%) if his performance is recognized under the same high demand-low control conditions. Analyzing this same scenario for women shows that the probability of stress falls by 38.5%, practically the same as for men. In conclusion, the sensitivity analysis shows that the influence that the recognition variable has on offsetting stress is high, as explained in the previous section, but similar in men and women.

Table 8 shows the stress probability values analyzed based on the age of the workers. Once more, the data is considered for the two extreme situations. In the worst scenario of high demands and low control, we see that every age range exhibits a higher likelihood of stress as recognition diminishes, with the highest variations present in the first two segments, under 35 and 35–45 (43.6% and 50.9%, resp.), with this latter segment having the highest stress probability at 79.2%. In the most positive situation for the worker, however, the greatest variations take place in the two older age ranges, 45–55 and older than 55 (38.9% and 44.4%, resp.).

## 4. Discussion

The main contribution of this study is the application of the Bayesian network methodology to carry out a combined and predictive analysis of the influence of demands (family, emotional, and job demands), control, social support (from colleagues and superiors), and recognition on the likelihood that a worker will experience stress. The influence of gender and age on stress is also considered.

The study also provides a Europe-wide analysis for healthcare professionals by crafting a Bayesian network with the database from the 6th European Working Conditions Survey (EWCS) and considering only the responses from healthcare professionals. This Bayesian network allows us to extract the knowledge from said database and, thus, understand the factors that most influence the work-related stress that is experienced by these workers.

The Bayesian network proposed first analyzes the influence of emotional demands and family demands as variables that buffer the stress caused by work factors. The results of the study show that a low emotional demand has more of an effect on reducing the likelihood of stress than low family demands.

The study also focuses on the Job Demand-Control model by Karasek [25] to analyze the influence that job demands and control have on work-related stress. The results obtained from the Bayesian network corroborate the findings in previous studies, in which high demands and low control yield the highest rates of stress, while low job demands and good control create lower levels of stress in workers [45, 69].

The BN model also analyzes the influence of social support as an element to offset stress. In this case, although the findings of our study coincide with those of previous studies—that is, that social support lowers stress [27, 70–72]—we found differences in the dampening effect depending on whether the support was provided by colleagues or superiors. The results of this study indicate that the support of colleagues, in general, has less effect on reducing stress than if a worker receives support from his/her superior. In turn, the lack of social support from work colleagues raises stress levels less than the lack of social support from superiors. Another finding of this analysis is that stress is more impacted by the demand factor than by the control or social support factors, confirming the conclusions reached by Pelfrene et al. [73] in their study.

At the same time, the model proposed makes it possible to analyze the influence of the recognition that workers receive on the likelihood that they will suffer from stress. The results show that, in situations of high demand and low control, recognition clearly impacts stress, drastically reducing it. In the same situation, workers who do not receive recognition are more likely to suffer from stress. At the opposite extreme, that is, with low demands and high control, stress also is impacted by recognition, with a lack of recognition having a greater impact than its presence.

In general terms, women experience higher levels of stress than men [29, 74]. The results of sensitivity analyses, undertaken to examine the effect of recognition by gender, show equality between men and women. In other words, for both men and women recognition has very positive effects, and its absence also has negative repercussions, but increases and decreases in likelihood are practically the same for the two genders.

Age is a determining factor in the analysis of work-related stress. The study indicates the worst stress results in the following scenario: situations of high demand, low control, a lack of recognition, and workers between the ages of 35 and 55. In this scenario, recognition plays a very important role, especially in those between 35 and 45 years of age, and is able to reduce the probability of stress by 50%. Another observable result is that younger workers are the most sensitive to recognition; until the age of 45 its effect is remarkable. By contrast, older workers, over 55, are not as sensitive to recognition; while it certainly helps to control their levels of stress, it does so to a lesser extent than in younger staff.

One of the findings of the study is the importance of emotional demands on work, as well as the support of colleagues and superiors and recognition for one's work. All of these aspects are reflected in the stress probability levels obtained. The detection of potential emotional implications for workers stemming from their jobs is an aspect that must be detected in order to attempt to reduce the stress generated by the demands of the job.

Company/government managers must provide their employees with a cooperative and supportive work environment. The absence of such an environment is a determining factor in the increased probability of suffering from occupational stress and in the adverse physical consequences on the health of affected workers.

Along these lines, if companies and government agencies were to recognize the work done by employees, this would go a long way toward reducing the likelihood of stress, lowering the workers' perceived workload and improving their occupational health. This recognition is particularly effective in middle-aged workers between the ages of 35 and 55, where the necessary stability of the worker is diminished due to the reasons stated. Recognition is also an important variable in the female workpool, for whom this aspect is more critical to the probability of suffering from occupational stress and its subsequent consequences.

The main limitation of our study involves the questionnaire of standard questions of the sixth European Working Conditions Survey; in other words, the variables used in our study had to be adapted to this questionnaire. In general, many of the variables in our study were built from the questions in the 6th EWCS, and not from the questions in the classical Karasek, ERI, and other models.  Table 2 shows the differences between the questions in classical models and the survey questions used in our study. For example, the “stress” variable defined by the health problems described in Q78 does not include gastrointestinal problems or appetite loss. A second limitation of the study is that Cronbach's alpha of the “stress” and “control” variables is low. Lastly, the sample size is a significant limitation to conducting a country-level analysis. For example, there are countries with fewer than 100 data points, which is not enough to train a model for each particular country. The same problem arises if we want to do an analysis by job type, meaning that there are not enough data points to itemize the study by job type.

Finally, we note the recommendations for future research. On the one hand, this study provides a stepping stone that can be used to further analyze some of the factors considered in future, more specific studies. On the other hand, a very interesting analysis would be to extend the methodology shown to the national scale, using national workplace condition surveys and breaking down the results for the different jobs involved in each sector, for example. Finally, an analysis over time that includes the ECWS for several years would provide some insight into how changes in working conditions affect the probability that work stress will occur.

In summary, based on our findings, those companies and government agencies with high occupational requirements should ensure that their workers have the social support of their superiors and implement measures to recognize the work of employees and minimize any potential emotional demands. This should lead to a lower probability of suffering from occupational stress and improve the quality of life and health of workers.

## Figures and Tables

**Table 1:** Level of education of the respondents.

	Frequency	Percentage
Early childhood education	1	0.05%
Primary education	5	0.23%
Lower secondary education	58	2.62%
Upper secondary education	554	25.06%
Postsecondary nontertiary education	203	9.18%
Short-cycle tertiary education	412	18.63%
Bachelor's or equivalent	515	23.29%
Master's or equivalent	362	16.37%
Doctorate or equivalent	101	4.57%

Total	2211	100.00%

Source: 6th EWCS data.

**Table 2:** Comparison of the questions in the main models.

	BN model	JCQ (Karasek et al. 1998)	Effort-Reward Imbalance (ERI) instrument (Peter and Siegrist 1999)	Job Demand and Resources (JD-R) (Demerouti et al. 2001)	Job demands: psychological job demands, physical demands, and emotional demands (De Jonge et al. 1999)	Copenhagen Psychosocial Questionnaire (Pejtersen et al. 2010)
Stress	“Did you have any of the following health problems? hearing problems, skin problems, backache, muscular pains, headache, anxiety and overall fatigue”				Psychosomatic health complaints, that is, “Do you have trouble with a headache in the last six months?”	

Emotional demands	“Please tell me, using the same scale, does your main paid job involve being in situation that is emotionally disturbing for you?”				Emotional demands: “In my work, death, sickness or human suffering are very demanding”	

Family demands	“How often in the last 12 months have you found it difficult to concentrate on your job because of your family responsibilities?”					“Do you feel that your private life takes so much of your energy that it has a negative effect on your work?”

Job demands	“Does your job involve working at very high speed?” “Does your job involve working to tight deadlines?”	2. Psychological job demands “Work fast” “No excessive work,” “enough time”	ER4 “I am often pressured to work overtime” ERI1 “I have constant time pressure due to heavy work load”	Job demands 2-time pressure: “I always have enough time to perform my task”	Psychological job demands: “In the unit where I work, work is carried out under pressure of time”	

Control	“Are you able to change your order of work?” “Are you able to choose or change your speed or rate of work?”	1b. Decision authority “A lot of say” “Little decision freedom”		Job resources 3-job control: “I can decide myself how to performer my work” Job resources 4-participation in decision-making: “Only the management decides what everybody has to do”	Job autonomy to determine a variety of task elements, like the method of working, the pace of work, and the work goals	

Social support from colleagues	“Your colleagues help and support you?”	3b. Coworker social support “Coworkers helpful,” “coworkers interested in me”	ERI 8 “I receive the respect I deserve from my colleagues”			

Social support from the boss	“Your manager helps and supports you?” “Your direct supervisor respects you?” “Your direct supervisor gives you praise and recognition when you do a good job?” “Your direct supervisor is successful in getting people to work together?” “Your direct supervisor is helpful in getting the job done?” “Your direct supervisor provides useful feedback on your work?” “Your direct supervisor encourages and supports your development?”	3a. Supervisor social support “Supervisor concerned” “Hostile supervisor” “Skills valuable” (5) “Coworkers work together” (3b) “Helpful supervisor” “Supervisor pay attention” “Career possibilities” (5)	ERI 9 “I experience adequate support in difficulty situations” ERI 7 “I receive the respect I deserve from my superiors” ERI 16 “Considering all my efforts and achievements, my work prospects are adequate”	Job resources 6-supervisor support: “My supervisor keeps distance from his/her employees” Job resources 1- performance feedback: “I get enough feedback about the quality of my performance”		

Recognition	“Do you agree or disagree with the following statement about your job. I receive the recognition I deserve for my work”		ERI 15 “Considering all my effort and achievements, I receive the respect and prestige I deserve at work” ERI 10 “I am treated unfairly at work”	Job resources 2- rewards: “My performance is rewarded property”		

Source: compiled by authors.

**Table 3:** List of selected variables and frequencies.

Group	Variable	States	*N*	%
Stress	Stress	Yes	814	36.8
		No	1397	63.2

Demographic	Gender	Male	465	21.0
		Female	1745	78.9
		No reply	1	.0
	Age	<35	590	26.7
		Between 35 and 45	533	24.1
		Between 45 and 55	644	29.1
		>55	444	20.1

Demands	Emotional demand	All & almost all of the time	565	25.6
		Around half of the time	832	37.6
		Almost never & never	811	36.7
		NR	3	.1
	Family demand	Always & most of the time	443	20.0
		Sometimes	691	31.3
		Rarely & never	1055	47.7
		NR	22	1.0
	Job demand	All & almost all of the time	433	19.6
		Around half of the time	898	40.6
		Almost never & never	870	39.3
		NR	10	.5

Control	Control	Yes	1518	68.7
		No	659	29.8
		NR	34	1.5

Social support	Social support of colleagues	Always & most of the time	1739	78.7
		Sometimes	238	10.8
		Rarely & never	102	4.6
		NR	132	6.0
	Social support of boss	Strongly & tend to agree	1314	59.4
		Neither agree or disagree	498	22.5
		Tend to disagree & strongly disagree	137	6.2
		NR	262	11.8

Recognition	Recognition	Strongly agree & tend to agree	578	26.1
		Neither agree or disagree	1240	56.1
		Tend to disagree & strongly disagree	373	16.9
		NR	20	.9

Source: compiled by authors.

**Table 4:** Sensitivity analysis: job demand, family demand, and emotional demand/stress.

Job demand	Stressed%	Family demands	Stressed%	Emotional demands	Stressed%
All & almost all of the time	51.49%	Always & most of the time	52.29%	All & almost all of the time	61.06%
		Rarely & never	49.15%	Almost never & never	37.37%

Almost never & never	29.26%	Always & most of the time	40.95%	All & almost all of the time	39.90%
		Rarely & never	26.35%	Almost never & never	21.20%

**Table 5:** Sensitivity analysis towards job demand-control-social support/stress.

Job demand	Control	Stressed%	Social support of colleagues	Stressed%	Social support of boss	Stressed%
All & almost all of the time	Yes	50.80%	Always & most of the time	49.85%	Strongly & tend to agree	45.25%
			Sometimes	57.24%	Neither agree or disagree	59.95%
			Rarely & never	60.87%	Tend to & strongly disagree	74.42%
	No	52.77%	Always & most of the time	49.35%	Strongly & tend to agree	45.04%
			Sometimes	69.33%	Neither agree or disagree	61.80%
			Rarely & never	73.55%	Tend to & strongly disagree	94.05%

Around half of the time	Yes	36.02%	Always & most of the time	36.55%	Strongly & tend to agree	31.98%
			Sometimes	41.88%	Neither agree or disagree	42.61%
			Rarely & never	34.63%	Tend to & strongly disagree	58.37%
	No	38.84%	Always & most of the time	37.69%	Strongly & tend to agree	32.23%
			Sometimes	49.41%	Neither agree or disagree	46.46%
			Rarely & never	41.72%	Tend to & strongly disagree	69.71%

Almost never & never	Yes	28.56%	Always & most of the time	29.84%	Strongly & tend to agree	26.16%
			Sometimes	32.70%	Neither agree or disagree	40.53%
			Rarely & never	26.71%	Tend to & strongly disagree	28.16%
	No	30.72%	Always & most of the time	31.66%	Strongly & tend to agree	27.40%
			Sometimes	31.76%	Neither agree or disagree	40.90%
			Rarely & never	21.93%	Tend to & strongly disagree	45.57%

**Table 6:** Sensitivity analysis towards job demand-control-recognition/stress.

Job demand	Control	Stressed%	Recognition	Stressed%
All & almost all of the time	Yes	50.80%	Strongly & tend to agree	35.76%
			Neither agree or disagree	48.28%
			Tend to & strongly disagree	69.24%
	No	52.77%	Strongly & tend to agree	30.68%
			Neither agree or disagree	52.57%
			Tend to & strongly disagree	69.45%

Around half of the time	Yes	36.02%	Strongly & tend to agree	24.36%
			Neither agree or disagree	34.73%
			Tend to & strongly disagree	59.56%
	No	38.84%	Strongly & tend to agree	25.97%
			Neither agree or disagree	35.57%
			Tend to & strongly disagree	63.58%

Almost never & never	Yes	28.56%	Strongly & tend to agree	21.16%
			Neither agree or disagree	28.64%
			Tend to & strongly disagree	51.25%
	No	30.72%	Strongly & tend to agree	17.89%
			Neither agree or disagree	31.73%
			Tend to & strongly disagree	50.68%

**Table 7:** Sensitivity analysis of job demand-control-recognition/stress & gender.

Job demand	Control	Stressed%	Recognition	Stressed%	Yes% Male	Yes% Female
All & almost all of the time	No	52.77%	Strongly & tend to agree	30.68%	20.71%	32.69%
			Neither agree or disagree	52.57%	40.29%	54.76%
			Tend to & strongly disagree	69.45%	58.60%	71.21%

Almost never & never	Yes	28.56%	Strongly & tend to agree	21.16%	17.24%	22.60%
			Neither agree or disagree	28.64%	22.41%	30.64%
			Tend to & strongly disagree	51.25%	44.74%	52.97%

**Table 8:** Sensitivity analysis to job demand-control-recognition/stress & age.

Job demand	Control	Stressed%	Recognition	Stressed%	AGE			
					Low <35	Between 35 & 45	Between 45 & 55	Over >55
All & almost all of the time	No	52.77%	Strongly & tend to agree	30.68%	14.85%	28.36%	48.06%	52.97%
			Neither agree or disagree	52.57%	43.26%	54.58%	65.09%	38.03%
			Tend to & strongly disagree	69.45%	58.43%	79.23%	75.98%	63.90%

Almost never & never	Yes	28.56%	Strongly & tend to agree	27.07%	24.46%	17.73%	17.41%	27.07%
			Neither agree or disagree	28.41%	32.29%	22.97%	32.17%	28.41%
			Tend to & strongly disagree	40.18%	43.44%	56.61%	61.81%	40.18%
